# A Proxy Design to Leverage the Interconnection of CoAP Wireless Sensor Networks with Web Applications

**DOI:** 10.3390/s150101217

**Published:** 2015-01-09

**Authors:** Alessandro Ludovici, Anna Calveras

**Affiliations:** 1 Wireless Network Group (WNG), Department of Telematics Engineering, Universitat Politècnica de Catalunya, C/Jordi Girona 1-3, Mòdul C3, Barcelona 08034, Spain; E-Mail: alessandro.ludovici@gmail.com; 2 i2CAT Foundation, Gran Capità 2-4 (Nexus Building), Barcelona 08034, Spain

**Keywords:** CoAP, WSNs, WoT, Smart Cities, 6LoWPAN, HTTP long-polling, low energy, WebSocket

## Abstract

In this paper, we present the design of a Constrained Application Protocol (CoAP) proxy able to interconnect Web applications based on Hypertext Transfer Protocol (HTTP) and WebSocket with CoAP based Wireless Sensor Networks. Sensor networks are commonly used to monitor and control physical objects or environments. Smart Cities represent applications of such a nature. Wireless Sensor Networks gather data from their surroundings and send them to a remote application. This data flow may be short or long lived. The traditional HTTP long-polling used by Web applications may not be adequate in long-term communications. To overcome this problem, we include the WebSocket protocol in the design of the CoAP proxy. We evaluate the performance of the CoAP proxy in terms of latency and memory consumption. The tests consider long and short-lived communications. In both cases, we evaluate the performance obtained by the CoAP proxy according to the use of WebSocket and HTTP long-polling.

## 1. Introduction

The adoption of the Internet Protocol (IP) in Wireless Sensor Networks (WSNs) is playing a major role in the realization of the Internet of Things (IoT) vision [1]. The integration of these technologies is changing the classical idea of the Internet that interconnects end-users to an Internet where physical objects can communicate with each other or with humans. The possibilities opened by this innovation have virtually no limitation in the fields where they can be applied.

The smart energy grid, building and home automation, e-health, Smart Cities and intelligent transport systems are only a few examples of application domains that would benefit.

The multiple applications of the IoT are attracting more and more interest from both the research and industrial communities. Organizations such as the IP for Smart Objects alliance (IPSO) [2] are promoting the use of IPs in embedded devices. Furthermore, the Internet Engineering Task Force (IETF) has standardized the use of the IPv6 protocols in WSNs. The resulting protocol stack is known as IPv6 over Low power Wireless Personal Area Networks (6LoWPAN) [3]. 6LoWPAN enables the transmission of IPv6 datagrams over low-power networks based on the IEEE 802.15.4 standard [4].

However, enabling IP networking alone in WSNs would not allow the potential of the IoT to be fully realized. At present, Web applications rely on the Hypertext Transfer Protocol (HTTP) to access information or to exchange data. The HTTP protocol is based on the Representational State Transfer (REST) architecture [5]. The adoption of REST in WSNs would ease the deployment and diffusion of IoT applications. Many existing applications would be reusable in this new environment. Furthermore, the integration of WSNs with the Web would be less complex and based on a mature technology. This new approach is referred to as the Web of Things (WoT).

The adoption of the REST architecture in WSNs has to be tailored to the constrained resources that characterize these networks. Typical WSNs nodes are battery-powered and equipped with a few kilobytes of memory and CPUs with reduced processing power. The impact of HTTP on these resources could be dramatic. An IETF working group called Constrained RESTful Environments (CoRE) [6] has been created with the aim of contributing to the development and standardization of the REST architecture in constrained networks. This working group defined a new Web transfer protocol called Constrained Application Protocol (CoAP) [7]. CoAP seeks to apply the same application transfer paradigm of HTTP to constrained networks, while maintaining a simple design and low overhead. CoAP implements a subset of the HTTP features and adds its own to best adapt to Machine to Machine (M2M) applications. With CoAP, we can manage applications and services offered by municipalities to citizen. In this sense, web services are of huge importance.

6LoWPAN and CoAP allow the IoT and Web worlds to be closer than ever. However, they are still too different to be easily integrated and interconnected. IoT and the Web, in fact, have different physical characteristics and are based on similar but different standards. In this sense, a Web application would still use HTTP and IPv6 to access an IoT device instead of using CoAP and 6LoWPAN. 6LoWPAN, in fact, is a standard thought to add IP capabilities to WSN and exploit IPv6 characteristics such as neighbor discovery and the large address space. However, the difference between the network links used in IPv6 and 6LoWPAN architectures requires the presence of a gateway to enable end-to-end communication between two endpoints using these protocols. The use of CoAP or HTTP-CoAP stack are not globally used. Therefore, it is of paramount importance the presence of systems able to interconnect Web applications with IoT devices.

**Figure 1.**
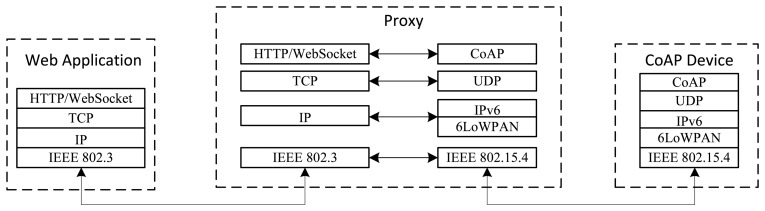
Protocol Stack. The Constrained Application Protocol (CoAP) proxy allows for adapting the protocol stacks of Web applications and CoAP devices.

**Figure 2.**
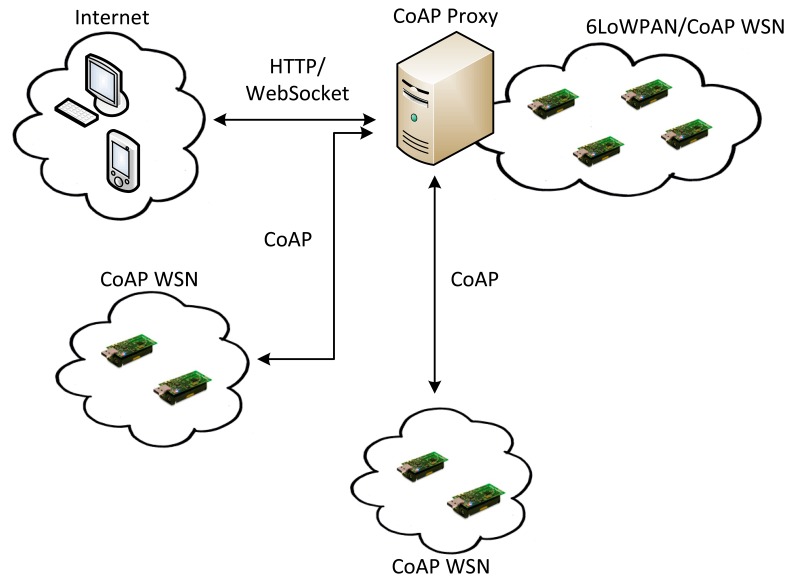
Network architecture. The CoAP proxy also has the IPv6 over Low power Wireless Personal Area Networks (6LoWPAN) edge router and gateway functions to interconnect disjointed CoAP networks.

In this paper, we present the design of a CoAP proxy. It enables Web applications to transparently access the resources hosted in IoT devices based on CoAP, which it is referred to as CoAP devices. Its main function is to adapt the different protocol stacks used by Web applications and CoAP devices (Figure 1). The CoAP proxy is designed to be located at the border of the 6LoWPAN WSN containing the CoAP devices, which enable the proxy to work also as 6LoWPAN edge router of the WSN. Moreover, this location enables the proxy to perform the function of CoAP gateway to interconnect disjointed CoAP networks. A graphical representation of the network architecture in which the CoAP proxy can be used is shown in Figure 2.

The CoAP proxy is designed to support applications that need to interact with WSN nodes, like Smart Cities developments. Traditionally, the HTTP long-polling technique has been used in these applications. However, it could result inefficient in these scenarios. The use of HTTP long-polling, in fact, forces Web applications to query constantly the CoAP proxy to receive data from the WSN. This could cause an excessive communication overhead and a consequent increase of latency and network traffic [8]. To overcome these problems, we include the WebSocket protocol [9] in the CoAP proxy design. WebSocket aims at providing a bidirectional communication channel using a single TCP connection, which allows the CoAP proxy to efficiently support long-lived communications. To ensure compatibility with the largest number of Web applications, the CoAP proxy also supports HTTP long-polling. Furthermore, the availability of a dual HTTP long-polling/WebSocket stack allows the CoAP proxy to adapt to different application requirements, which is a key aspect in Smart City applications.

We demonstrate the effectiveness of our design by a performance evaluation in a real WSN. This evaluation is performed in terms of latency and memory consumption. The CoAP proxy is evaluated considering long and short lived communications established between the Web application and a CoAP device. In short-lived communications the Web application requests to the CoAP proxy a resource hosted in the CoAP device. The proxy replies immediately with the representation of the resource. In this case, the communication ends after the Web application receives the response. Long-lived communications are used when the Web application needs to be notified about the changes of a resource over the time. In this case, the transmission is continuous and ends only when one side of the communication explicitly closes it. In both situations, the performance of the CoAP proxy is evaluated according to the protocol used by the Web application to access the WSN.

There is little work in the literature about the design of a CoAP proxy. The authors of [10] describe the main building blocks of a simple CoAP gateway used to monitor remotely a WSN. The gateway performs as a cross-proxy to translate HTTP requests into CoAP ones. The use of a 6LoWPAN gateway to monitor a CoAP WSN is also presented in [11]. The authors of this paper design a notification system to achieve the interaction between the Web application and the gateway. However, these works do not consider the case where the monitoring could be long-lived and do not present any performance evaluation.

The CoAP proxy presented in this paper is designed to provide support to applications that need to continuously retrieve data from the WSN. The main contribution of this paper is referred to the design and implementation of a proxy able to interconnect Web applications to CoAP devices located in WSNs, as ones conformed by Smart Cities M2M architectures. The presence of a CoAP proxy is of paramount importance to interconnect HTTP based networks with CoAP WSNs. It is expected, in fact, that most of the accesses to CoAP WSNs will come from traditional HTTP networks.

The rest of the paper is organized as follows. First, in the following section we discuss the design considerations. The design of the proxy is illustrated in Section 3. Results and discussion of the performance evaluation are reported in Section 4. Finally, Section 5 concludes the paper.

## 2. Smart City and Web Applications

Smart Cities are an emerging research field with many different proposals in the latest years. Several services have been envisioned and new ones are constantly appearing, but the ideal network architecture to be used has not been envisioned yet. The Smart City concept is one of the applications IoT concept. Therefore, that, Cities can be seen as a number of “things” that allow better municipality management, citizens interactions with its municipalities by the informational services, health, traffic congestion, parking places, light presence based in the streets and parks, pollution indication, *etc*.

M2M communications are the key to allow the Smart City concept to become a reality. In Smart City platforms sensors, actuators and system information perform a WSN.

Building a Smart City implies the assurance of the connectivity of a lot of sensors and actuators, and transferring data in a M2M manner. In some cases, reliability, and always scalability are essential features. Nodes that composes a Smart City architecture mainly are resource-constrained devices, therefore protocols have been adapted to them. IPv6 has been adopted to interconnect these networks. Specific wireless technologies such as IEEE802.15.4 are used to rapid, cost efficient and energy sustainable deployments. The adopted architecture for these networks is shown in Figure 2 and protocols introduced in Section 1.

The used sensors and actuators are very wide and they are only limited by the applications such as lighting control to monitor the brightness level of the street and turn on or off street lighting; climatic conditions as temperature, humidity, atmospheric pressure, wind and rainfall; green zones irrigation control; monitoring of air pollution for measuring the concentration of certain gases such as CO_2_, SO_2_ or NO_x_; monitoring of noise pollution; public safety by means of presence of people using for instance PIR (Passive InfraRed) devices; light level or noise level measurements; underground infrastructure monitoring present in a city like pipelines, telecommunications, water, effluent, gas or electricity; information on traffic congestion to plan travel time and route; city updated information on the presence of parking; rubbish containers motorization to optimize recollection, *etc*.

All these devices that conform a Smart City deployment are constrained devices and must be accessed and actuated from different control network elements. As stated in [7], one of the main goals of CoAP is to design a generic web protocol for the special requirements of this constrained environment, especially considering energy, building automation, and other M2M applications which are normally used in Smart Cities developments. With CoAP we can manage applications and services offered by municipalities to citizens. In this sense, web services are of huge importance.

Web applications for smart cities depends usually on the Internet reliable HTTP protocol, which has not been designed for the constrained networks. Therefore, it introduces overhead, wastes energy and in some cases cannot be implemented in cheap and simple devices. To overcome these issues the use of a CoAP proxy is capable of using an optimized scenario in the sensor and actuators networks based on CoAP protocol stack, and traditional web applications to access and actuate these resources.

The introduction of the CoAP proxy makes it possible to interconnect constrained networks to traditionally Internet ones at the application layer, being transparent to the user applications (in terms of network semantics). Therefore, we can take maximum advantage of web applications applied to the deployment of Smart Cities, maintaining the optimization of constrained devices requirements.

**Figure 3.**
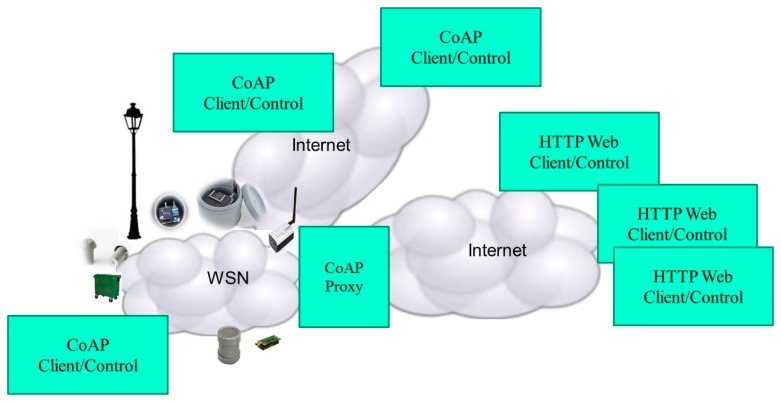
CoAP and HTTP access and actuation in a Smart City environment.

Figure 3 presents a Smart City scenario where sensors and actuators are interconnected either by HTTP web based applications or CoAP ones.

Smart City web applications are mainly HTTP based, they do not use the CoAP client, and then they need the CoAP Proxy, an element to interconnect sensors and actuators from these application services. Without this element, a large number of applications cannot be connected to the offered resources and the interaction with actuators will not be possible. In conclusion, the use of the CoAP or HTTP-CoAP stack is not global. Therefore, it is of paramount importance the presence of systems able to interconnect Web applications with IoT devices. Therefore, to realize an “end-to-end” Web application environment in a Smart City scenario that can support a wide range of M2M applications, the translation of HTTP to CoAP or CoAP to HTTP are required.

## 3. Design Considerations

A basic consideration that affects most of the design decisions relates to the communication patterns being used. Should the proxy communicate with a Web application, the communication pattern has to consider the possibility that the data exchange could be long-lived. In this case, traditional communication patterns such as the HTTP long-polling could be inefficient. Instead, the interaction between the CoAP proxy and a CoAP device has to consider the limited bandwidth that characterizes the wireless link. In this case, a request/response model could lead to a wasteful use of bandwidth. The rest of this section focuses on the design considerations for the communication patterns used by the CoAP proxy to communicate with the CoAP device and the Web application. Furthermore, we discuss the translation process followed by the CoAP proxy to map between the URIs [12] used by Web applications and CoAP.

### 3.1. Communication Pattern between the CoAP Proxy and the CoAP Device

The information flow of a WSN is mainly constituted by data collected by sensors, as in a Smart City environment. Data can be collected periodically or in occurrence of an event. In either case, it has to be transferred elsewhere in the WSN or to external networks. The WSN can be composed by a large number of nodes and could interact with many external networks. The data transfer model to adopt should, therefore, be compliant with these characteristics. The traditional request/response model requires us to periodically poll the CoAP device to receive updates on the monitored resource or event. As a consequence, the traffic could increase congesting and overloading the WSN. Furthermore, the resource consumption would grow. Instead, an asynchronous model would be more suitable in these situations. The CoAP device would transfer data only when these are available and not in response to a request.

**Figure 4.**
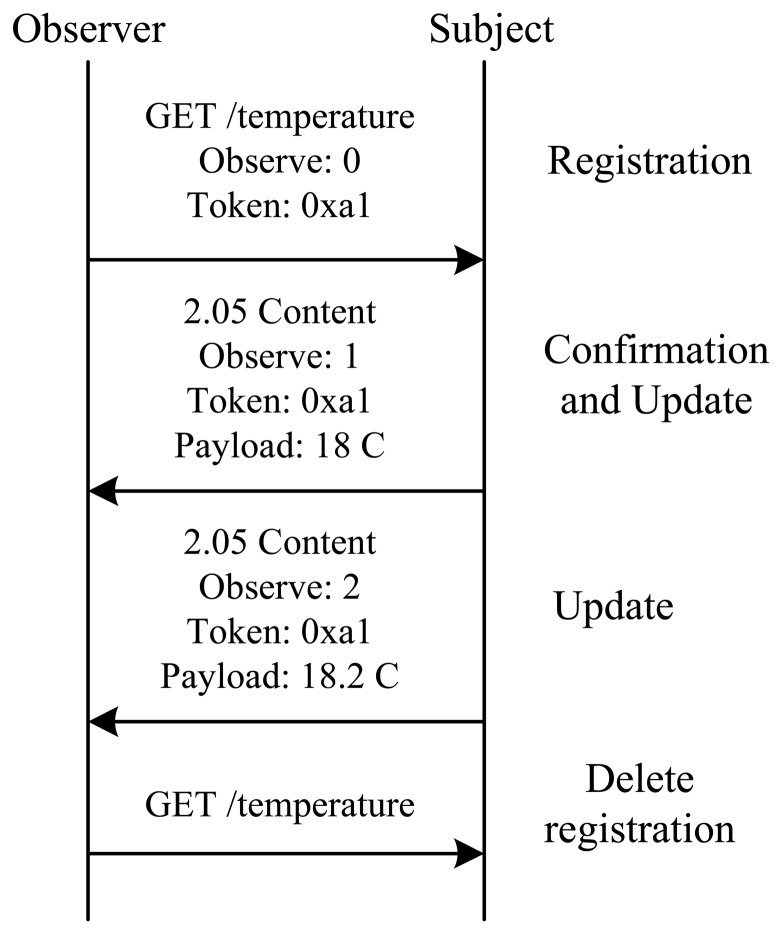
Observe protocol. The observer deletes its registration after receiving two updates.

CoAP implements this asynchronous data-exchange model trough the observe protocol [13]. This implements the well-known observer design pattern [14]. The observe protocol defines two components: the observer and the subject. The observer is a client interested in being updated with changes of the resource state while the subject is the server that provides the updates. The client registers as an observer by sending a CoAP GET request message containing the observe option to the subject hosting the resource of interest. As a consequence, the subject registers it as an observer and then notifies it when the resource state changes. To confirm the successful registration, the subject sends a response with the current resource state and the observe option. The value of the observe option is used to order chronologically the updates. An observer has two options to end the observe relationships: it could respond to an update with a RST message or it could send a simple GET request message to the observed resource. Figure 4 shows an observer registering to a resource, receiving an update and then deleting its interest.

The CoAP proxy uses the observe protocol to receive updates from the CoAP device. In our design, the CoAP proxy is the only observer registered to the CoAP device. Web applications establish the observe relationship only with the proxy. Consequently, the WSN network traffic is significantly reduced allowing for minimizing the bandwidth usage of the wireless link. Moreover, the CoAP device is subject to less work and it is able to minimize the resource consumption. The observe relationship between the CoAP proxy and the CoAP device is established when the first Web application requests it.

### 3.2. Communication Pattern between the Web Application and the CoAP Proxy

The CoAP proxy is designed to support long-lived transmission of data. This enables the proxy to be used in WSN applications such as industrial monitoring, e-health or Smart Cities. HTTP has not been originally designed to work with long-lived applications. However, techniques that simulate this behavior can be applied. In this sense, HTTP polling has been largely used to support long-lived communications over the Web. In this technique, the Web application sends periodical requests to obtain the data. If these were not available it would receive an empty response from the server. Polling is only suitable when the message delivery interval is constant and known. Furthermore, it should be long enough to ensure that the overhead would not increase latency or network traffic. A more efficient solution is provided by HTTP long-polling. In HTTP long-polling the server holds the client request until new data is available or the TCP timeout expires. This solution reduces significantly the number of useless messages that are interchanged in HTTP polling. Although HTTP long-polling solves some of the problems that affect HTTP polling, the overhead inherent to sending periodical HTTP requests still remain unsolved.

An effective solution for long-lived communications is provided by the WebSocket protocol. This protocol enables establishing full-duplex and bidirectional communications over a single TCP socket. With WebSocket, a Web application can receive data from the CoAP proxy avoiding establishing multiples HTTP connections. When new messages are available, the CoAP proxy sends them over the existing connection.

A WebSocket communication has three phases: the opening and closing handshakes and the data transfer. A WebSocket connection can be initiated only after a TCP connection has been established. A client sends a WebSocket handshake request to initiate the opening handshake. This is equivalent to an HTTP upgrade request as specified in the Upgrade and Connection header fields of the handshake request. The request message contains the version of the protocol and the hostname of the server. The handshake request contains also the HTTP method and the URI of the resource as shown in the following example:

GET /temperature HTTP/1.1
Host: proxy.com
Upgrade: Websocket
Connection: Upgrade
Sec-WebSocket-Key: x3JJHMbDL1EzLkh9GBhXDw==
Sec-WebSocket-Version: 13

To establish a WebSocket connection, the server has to prove to the client that it received the handshake request. To provide this proof, the server concatenates the content of the Sec-WebSocket-Key header field with a Globally Unique Identifier (GUID) [15]. The server returns the hash of this concatenation in string form in its handshake response. This is contained in the Sec-WebSocket-Accept header field as shown in the following example:

HTTP/1.1 101 Switching Protocols
Upgrade: Websocket
Connection: Upgrade
Sec-WebSocket-Accept: HSmrc0sMlYUkAGmm5OPpG2HaGWk=

The server response includes also an HTTP status line. The WebSocket connection is only established when the status code is 101. The Upgrade and Connection fields are used to complete the HTTP upgrade.

**Figure 5.**
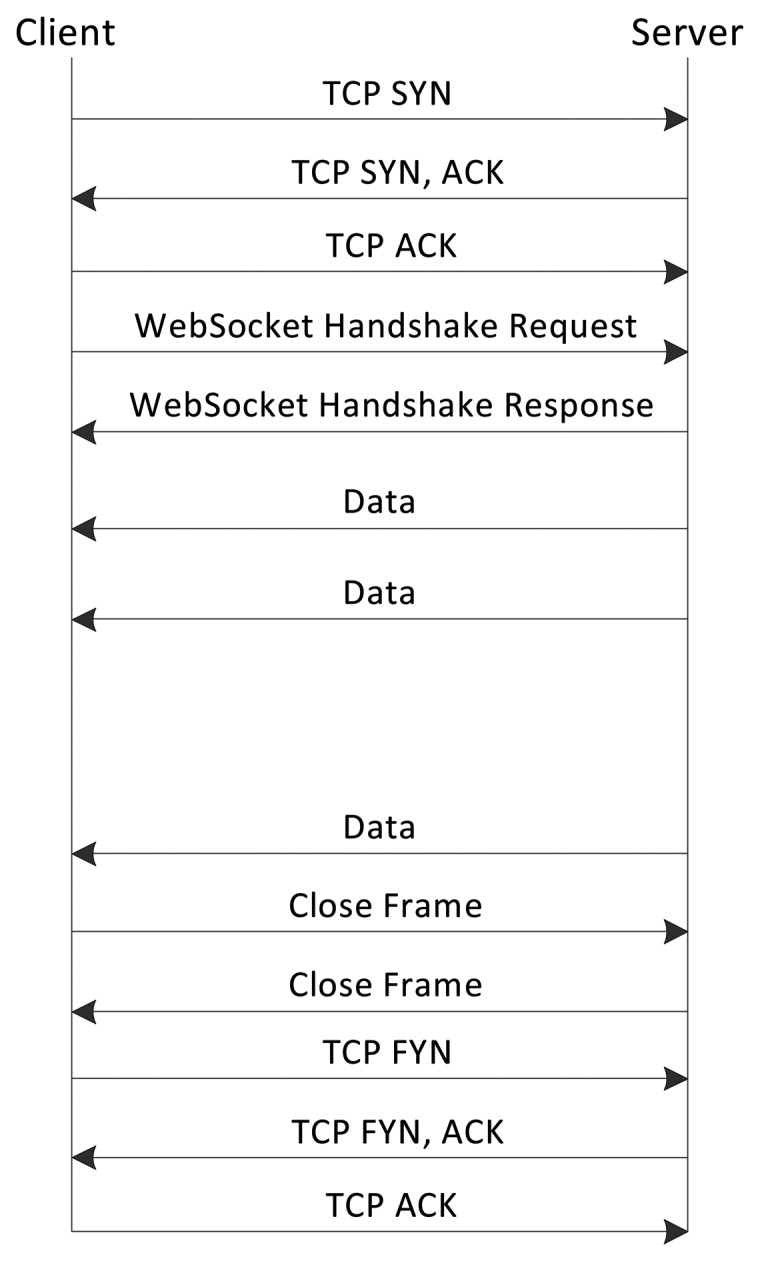
WebSocket protocol. The WebSocket communication consists of an opening handshake, a data transfer and a closing handshake.

Should the handshake phase be successful, the bidirectional channel is established and the data transfer phase can start. Data is sent in data frames defined by the WebSocket standard. The base data frame is formed by an option code, a payload length and data. The option code is used to interpret the data. A data frame can be sent either by the client or the server. The WebSocket connection is closed after a closing handshake. This can be initiated by any of the endpoints by sending a WebSocket close frame. The other endpoint also replies with a close frame. After the closing handshake, the endpoints close the TCP connection. Figure 5 shows the messages interchanged between a client and a server to establish a communication using the WebSocket protocol.

In short-lived communications, HTTP long-polling may have better performance than WebSocket. In HTTP long-polling, in fact, the interaction requires only the interchange of a HTTP request and a response message. Instead, in the WebSocket case, the overhead introduced by the handshake phases could increase latency.

### 3.3. Protocol Translation

Interconnecting Web applications and CoAP devices require providing a solution to translate the protocols involved in the communication. The methods, response codes and content-type supported by HTTP, WebSocket and CoAP are equivalent and allow a straightforward and transparent mapping. They all identify resources using the syntax defined by the URI standard. The path and authority parts are equivalent while the scheme varies depending on the protocol used. Our proxy supports the “http” and “ws” schemes defined by the HTTP and WebSocket protocols respectively. They are translated directly to the “coap” scheme. The authority part of the URI contained in the Web application request corresponds to the combination of the IP address and TCP port of the proxy. The authority part of the CoAP device that hosts the requested resource is derived after the translation process of the URI path, which identifies the resource target of the request.

At present, the CoRE working group is defining best practices for HTTP-CoAP mapping [16]. The document includes several proposals that seek to establish a common URI format to be used by HTTP request. However, these proposals do not define a shared format able to identify the targeted resource or the request to establish an observe relationship.

**Figure 6.**
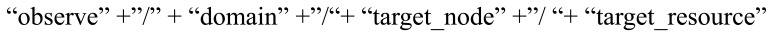
URI format.

In this paper, we proposed and adopted a novel format for the URI path. Its structure is derived from the Core Resource Directory (RD) [17] specifications. The RD is a Web links repository that allows for hosting of the information related to CoAP devices and the resources they expose. We include the RD repository on our proxy and use the information hosted in it to map the HTTP and the CoAP URI paths. We proposed and adopted the format showed in Figure 6.

The first part of the format is optional and indicates the willingness to establish an observe relationship. A short-lived request must be sent without the observe part. The domain is included to support WSNs with complex topologies. A domain is considered as a logical grouping of nodes [17]. For example, a WSN could be deployed over different floors of a building; in this case the nodes of the same floor can be grouped in the same domain. The domain part is optional. The name of the CoAP device and that of the requested resource are expressed as target_node and target_resource, respectively. A target_node is unique within a domain while a target_resource is unique in a target_node. As an example, considers a node, which is node_1, placed on the first floor of a city building that measures temperature and exposes it as a resource. The resulting URI path to observe this resource is shown as follows:

observe/floor_1/node_1/temperature

The complexity of the URI path composition is hidden from the Web application. It learns the URI performing a resource discovery on the CoAP proxy. CoAP defines a URI for this purpose which is called “.well-known/core” [7]. Any request directed to that URI results in a response containing the resources offered by the proxy.

**Figure 7.**
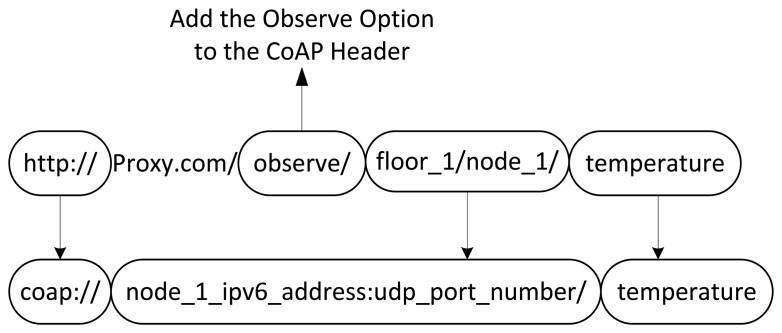
Translation of the HTTP URI into a CoAP one. The URI used by WebSocket has the same format of the HTTP URI except for the scheme. WebSocket used the “ws://” scheme.

The URI format, as shown in Figure 6, differs from that used by the CoAP proxy or by another node to interact directly with the CoAP device and query the same resource. The only part that is equivalent is the target_resource. In a direct interaction, the observe relationship is established including the observe option in the CoAP header and not in the URI path. Furthermore, the domain and target_node are replaced by the combination of the IPv6 address and UDP port of the server. The domain part could be introduced by the proxy and could not correspond to any composition of the real CoAP URI. The CoAP proxy translates the URI in Figure 6 into the equivalent used for direct interaction as shown in Figure 7.

## 4. Proxy Design and Implementation

A CoAP proxy can be classified as one of three categories depending on the role that it has in the network architecture [7]. In particular, a proxy is classified as a forward-proxy when it performs requests on behalf of the client. Instead, a proxy that behaves as if it were the real server is classified as a reverse-proxy. Finally, a proxy that translates between different protocols is defined as a cross-proxy.

In our design process, all of these roles are grouped inside the same proxy. They are, in fact, complementary and not opposite.

As an example, a reverse-proxy could be at the same time a cross-proxy since it may have to translate the received request. Furthermore, the concept of reverse and forward proxy can co-exist in the same device. In fact, these roles are services offered by the CoAP proxy more than strict categories. We implemented the reverse-proxy service to establish end-to-end data transactions between a Web application and a CoAP device. The forward-proxy is implemented to allow proxing operations inside the WSN. End-to-end connectivity between disjointed CoAP networks can be provided by both services.

Three modules that, at run-time, are executed in separate processes, compose the CoAP proxy. UNIX sockets are used to establish communication between them. The first module is the Lighttpd server. It is in charge of receiving the incoming HTTP long-polling requests. The 6LoWPAN interface, instead, allows the proxy to communicate with the WSN. Finally, the main proxy module implements the core functionalities provided by the CoAP proxy. This module is composed by the following components:

- CoAP module.
- Web server module.
- Cache.
- Resource Directory.

**Figure 8.**
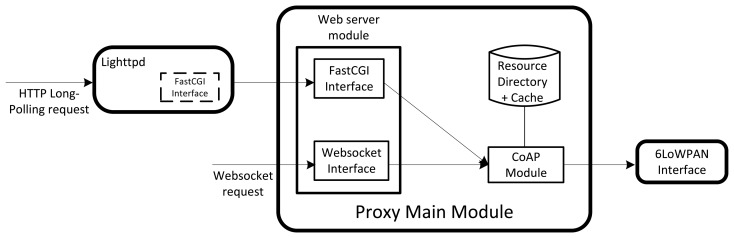
CoAP proxy design. The CoAP proxy is composed by three modules.

Figure 8 shows the composition of the proxy.

### 4.1. 6LoWPAN Interface

As previously mentioned, the CoAP proxy has also the function of 6LoWPAN edge router. This is in charge of forming the 6LoWPAN network and route between the 6LoWPAN network and other IP networks. The edge router provides the IPv6 network prefix to nodes. These nodes learn them the prefix using the 6LoWPAN neighbor discovery [18]. In this mechanism, a node sends a broadcast message at startup, which is called the router solicitation message. The edge router replies with a message containing the IPv6 network prefix, which the node uses to auto-configure its address. The node learns the address of the edge router from the same message.

In our implementation, the address of the edge router corresponds to that of the CoAP proxy. Therefore a CoAP device does not need to use any proxy discovery mechanism. This allows for reducing the number of messages interchanged by the CoAP device and the CoAP proxy and therefore saving the device's battery power.

The CoAP proxy is connected to the WSN via an 802.15.4 interface with a 6LoWPAN layer. 6LoWPAN functionalities are provided by Blip [19], which is also embedded in CoAP devices to enable IPv6 networking.

### 4.2. Lighttpd Module

As previously mentioned, we included WebSocket and HTTP long-polling in the CoAP proxy. The module responsible for handling WebSocket requests is integrated in the main proxy module while that of HTTP long-polling is split in two parts. In this sense, an HTTP server is in charge of establishing the HTTP connection while the main proxy module performs the relative URI translation process. There are several implementations of HTTP servers that already exist and are commonly used. Between these, we selected Lighttpd [20]. It has, in fact, a lower memory footprint and requires less CPU usage respect to other solutions. Both aspects are of paramount importance to reduce the resource consumption. The CoAP proxy, in fact, could be embedded in constrained hardware where the minimization of the resource consumption is essential.

### 4.3. Main Proxy Module

The main proxy module provides the core functionalities of the CoAP proxy. As mentioned before, this module includes the RD repository, cache memory and the CoAP and Web server modules. The translation process of the HTTP and WebSocket URIs to CoAP ones is implemented in this module. In particular, both the Web server and CoAP modules perform this process. The CoAP module is also responsible for handling the observe protocol and maintains the RD and cache memory. The Web server module includes the interfaces needed to receive WebSocket requests and to communicate with the Lighttpd server.

The composition and the functions of the main proxy module are detailed in the rest of this section.

#### 4.3.1. Web Server Module

The Web server module is one of the essential building blocks of the main proxy module. It allows Web applications to communicate with the CoAP proxy. Its main role is to establish the connection between them and to handle the incoming requests. HTTP long-polling and WebSocket are implemented as separate modules. As mentioned before, the Lighttpd module is responsible for receiving the incoming HTTP long-polling requests. The Web server module and Lighttpd interact using the FastCGI protocol [21]. The use of FastCGI has been preferred to that of the simple CGI protocol. FastCGI has better performance with respect to those of CGI and it is able to reduce the process overhead. It uses long-lived processes to handle multiple requests instead of creating new processes for each request as done by CGI.

The requests received by the Web server module are stored in a data structure, which is connection_t. This contains the information related to the connection established by the Web application with the CoAP proxy. Furthermore, connection_t stores the CoAP PDU resulting from the translation process of the URI. The connection_t structure is composed as follows:
typedef struct { int type; char *path; void *data; coap_pdu_t *pdu;} connection_t;

Type indicates the protocol used by the Web application. This could be Websocket or FastCGI if the connection has been established trough HTTP long-polling. Path contains the URI targeted by the request. Finally, the socket number of the connection is stored in data while pdu contains the CoAP message created after the translation process.

**Algorithm 1.** The Web server module receives a Websocket request, checks its validity and passes it to the CoAP module.
**Input**: Websocket request from Web client.**Output**: node entry from RD.**for** each incoming Websocket request **do** accept TCP connection; URI ← Websocket_do_handshake(socket); **if** URI contains “observe/” **then**  observe ← 1;  remove ”observe/” from URI; **end if** node_id ← get_node_id(URI); node_entry ← get_node_entry_from RD(node_id) connection ← create a connection_t structure; pass the requests to CoAP module (connection, node_entry, observe);**end**


The correct translation of the URI contained in HTTP and WebSocket messages requires checking its validity and correctness trough the analysis of the scheme and path parts. This can be considered as the first step of the translation process. At this level, it focuses on verifying the presence of the observe part in the URI and the existence of the domain and target_node in the RD. Instead, the validity of the observe request as well as the target_resource are verified by the CoAP module. This is subsequent to the validation of the target_node. The structure of the RD is presented and discussed in the next section. Algorithm 1 illustrates the procedure used by the Web server module to accept and validate a Websocket request. The algorithm used for handling HTTP long-polling request is similar except for the fact that the TCP and HTTP connection are established by the Lighttpd server. In this case, the Web server module receives the information about the Web application and the URI trough the FastCGI protocol.

#### 4.3.2. Resource Directory

Direct resource discovery using the “.well-known/core” URI is a useful mechanism only when the discovery is internal to the WSN. It becomes inefficient when a device or application external to the WSN wants to learn the resources hosted in its nodes. In this context, the use of an RD entity simplifies this task. The RD hosts the description of the resources provided by the WSN nodes. The resource description in CoAP is achieved using the Core link format [22] that is equivalent to the Web linking [23] used by HTTP. The WSN nodes are responsible for registering and keeping updated their entries in the RD. The RD provides the interfaces for registering, updating and deleting entries as well as for performing resource discovery. The entry point of the RD is the “.well-known/core” URI.

The RD hierarchically is organizes in such a way so that each node corresponds a single entry. The resources are linked to the corresponding node entry. In fact, a single node could offer several resources. Therefore, indexing the RD using resources instead of nodes could lead to an intricate structure that complicates the resource discovery and the translation of the URI. Using the node description as index allows simplify the RD structure and the operation that are performed on it. The node description includes the node name and type, the IPv6 address and UDP port and optionally the domain. The node name and domain correspond to the target_node and domain part of the URI as shown in Figure 6.

**Figure 9.**
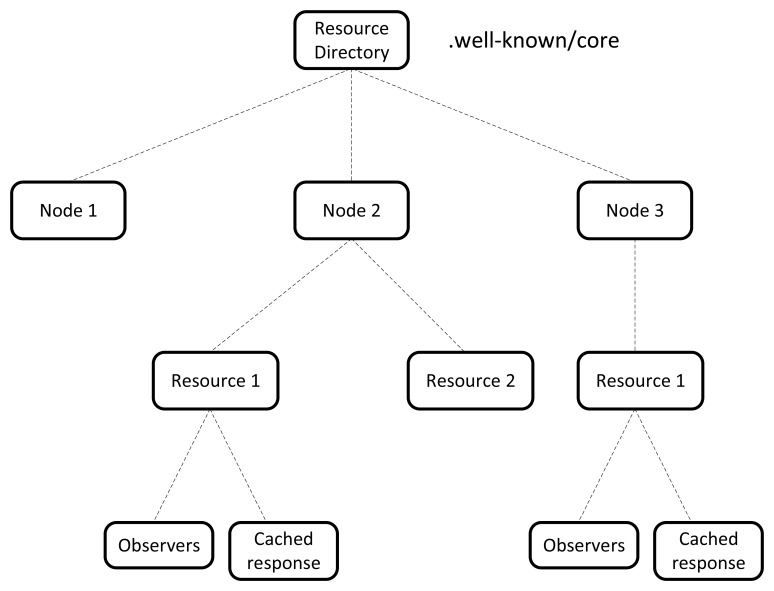
RD structure. The RD is designed as a tree-structure and it is indexed by the node description.

The RD is designed as a tree-structure where the node description is placed at the top and the resource descriptions are the branches of each entry. The resource description includes the path and the semantic type of the resource, the estimation of the payload size and a flag to indicate if it is observable or not. The resource path corresponds to the target_resource part of Figure 6. The RD structure is shown in Figure 9. To simplify the management of the resources we added an extra layer at the bottom of the resource description. The purpose is to store the lists of observers of each resource. The cached response is also linked to the corresponding resource entry. This allows simplifying the use of caching. It is, in fact, strictly related to the resource and separating its representation from the resource entry may complicate its use. Cache is explained in the next section.

#### 4.3.3. Cache

CoAP is a protocol specifically designed for constrained devices where the minimization of resource consumption is crucial. As a consequence, it is expected that some devices might be in sleep mode the majority of the time in order to save as much energy as possible. Furthermore, the bandwidth available in WSN links is limited. The reduction of the number of interactions between the CoAP proxy and the WSN is therefore necessary.

Under these conditions, a caching system is essential in the CoAP proxy. The presence of cache allows reducing the number of queries that the proxy sends to a CoAP device to obtain the state of a resource. CoAP defines a freshness and a validation model for caching [7]. A CoAP device indicates explicitly whether a response or an observe update is cacheable or not. Furthermore, it can indicate the expiration time of the cached response using the Max-age option. When the cached data is no more valid the proxy can ask the server to renew its validity. This is accomplished by using the ETag option. Caching is particularly useful for the observe updates. A CoAP device may use caching to avoid sending periodical updates containing unchanged data. Cached data could also be used to send the first notification to a new observer if the CoAP device has not sent a new update after its registration.

The CoAP proxy stores the payload of the response and a timestamp. The timestamp refers to the time at which the cached message has been received and it is used to check the validity of the cached payload. As previously mentioned, the cache is contained in the RD structure. The CoAP module is responsible for the management of the cache. Next section illustrates the design and functions of this module.

#### 4.3.4. CoAP Module

**Figure 10.**
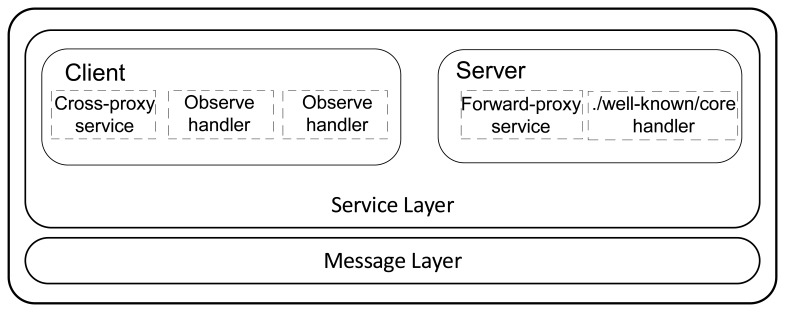
CoAP module overview.

As can be seen from Figure 10, the CoAP module is split in two layers. The message layer interacts with the 6LoWPAN interface allowing sending and receiving messages to and from the WSN. The functionalities needed to create and manipulate CoAP messages are also implemented in this layer. The service layer handles CoAP requests and responses. It is split further in two sub-modules: a client and a server. Both client and server operate on the RD and the cache with different purposes.

The functionalities of both sub-modules are described as follows.

##### (a) Client Sub-Module

The client sub-module provides the cross-proxy service. As previously mentioned, the operation performed by this service focuses on the translation between protocols. The result of this process is a CoAP message that matches the request of the Web application as specified in the connection_t structure, which is passed by the Web server module. The client's functions also include the management and establishment of observe relationships. The management of observe requests as that of simple requests is compliant with the characteristics of the reverse-proxy service. The client sub-module is also in charge of validating the cached responses and using its content to reply to a request.

The creation of a CoAP request message is subsequent to the validation of the resource targeted by the original request. As mentioned above, the domain and target_node parts of the URI are validated by the Web server module. Therefore, the client sub-module only checks if any resource linked to the target_node matches the resource specified by the target_resource. Should a match be found, the client sub-module verifies if the request is intended to establish an observe relationship. In that case, it checks that the CoAP device has labeled the resource as observable and, if positive, it adds the observe option to the CoAP message.

The client sub-module sends the observe request to the CoAP device only if an observe relationship with the same resource does not yet exist. This relationship is, therefore, established only when the first Web client requests it. The CoAP proxy is, in fact, the only observer registered directly at the CoAP device. Instead, the Web clients are registered only at the CoAP proxy. As a consequence, the CoAP device sends the updates only to the CoAP proxy that will distribute them to Web clients. This allows for reducing the resource consumption of the CoAP device and the bandwidth usage of the wireless link. A further implication of this choice is to increase the number of Web clients that can observe a resource. The wireless link of the WSN is, in fact, unable to sustain the high-traffic that is provoked by multiple observers. Moving this traffic to the more capable link between the Web clients and the CoAP proxy ensures the sustainability of a higher number of observers and the scalability of the solution. Algorithm 2 shows the translation process followed by the client sub-module. The CoAP request message is sent after verifying the existence of the resource and the validity of the observe request.

**Algorithm 2.** Translation process followed by the client sub-module.
**Input**: connection_t, node entry in RD, observe flag**Output**: CoAP request, observe request, cached responseresource ← get_resource_from_node_entry(connection_t)**if** resource does not exist **then** return error**if** observe == 1 AND resource is observable **then** **if** resource.observer_list[] is empty **then**  connection_t.pdu ← create CoAP request;  insert observe and URI options →connection_t.pdu;  send observe request to CoAP device;  coap_connection ← create a coap_connection_t structure;  coap_connection → pending_queue[];  connection_t → observer_list[]; **else**  connection_t → observer_list[]; **end if** **else**  **if** cached response is valid **then** send cached response to Web client  **else**   connection_t.pdu ← create CoAP request;   insert URI options → connection_t.pdu;   send request to CoAP device;   coap_connection ← create a coap_connection_t structure;   coap_connection → pending_queue[];**end if**


The CoAP request message and the information related to it are stored in the coap_context_connection_t structure. This allows the client sub-module to match the response received by the CoAP device with the requests it has sent. A linked list is used to implement the queue containing these structures (pending_queue). The coap_context_connection_t elements stored in this queue are indexed by the token value contained in the CoAP request. CoAP defines the use of tokens to match univocally requests with response. A response is subsequent to a CoAP request if it has the same token value. As shown in Algorithm 3, the client sub-module extracts the token contained in the response to find a match between the responses stored in the pending_queue. The list of observers of a resource is also implemented using a linked list. In this case, it contains the connection_t structure received by the Web module. The coap_context_connection_t is implemented as follows:
typedef struct { unsigned int n_retransmit; time_t timestamp; unsigned int is_observe; unsigned int is_separate_response; connection_t *connection;} coap_context_connection_t;

**Algorithm 3.** response_handler
**Input**: response from CoAP device**Output**: response message or observe update to Web clienttoken ← get token from response;coap_connection ← get_connection_from_pending_queue(token);**if** response is cacheable **then** update cache;**end if****if** coap_connection.is_observe == 1 **then** get observer_ list[] from the RD; **for** each observer in the observer_ list[] **do**  send observe update;  **if** send observe update fail **then** remove observer from observer_list[]; **end for****else** send response to Web client; remove coap_connection from pending_queue[];**end if**


The n_retransmit and timestamp fields are used by the CoAP retransmission mechanism, which follows a stop-and-wait model with exponential back-off. In particular, timestamp stores the time at which the message has been sent and it is used to calculate the value of the back-off. The number of retransmissions is maintained by the n_retransmit counter. The connection pointer is used to access the CoAP message and the information on the request received by the Web client, which are contained in the connection_t structure. The is_observe and is_separate_response flags are used to specify the kind of the data transaction established with the CoAP device. The is_separate_response flag indicates that the CoAP device will not reply immediately to the request. CoAP defines a separate response mechanism to enable devices to delay the reply if they cannot process the request directly.

##### (b) Server Sub-Module

The server sub-module provides the forward-proxy service. This enables CoAP devices belonging to the same WSN to interact with each other through the CoAP proxy. This service is particularly useful when they are several hops away from each other and direct interaction could squander precious resources. The forward-proxy service is requested including the Proxy-URI option in the CoAP request message. The server sub-module can forward the request to the destination or reply with a valid cached response. The forward-proxy service can also be used to observe a resource trough the CoAP proxy.

The server sub-module provides the interface required to handle queries directed to the “.well-known/core” URI and, consequently, to manage the RD. It also initializes the cache memory for a given resource. The initialization is subsequent to the creation of a new resource inside the RD. The client sub-module is, instead, responsible for validating and updating the cache, as well as for replying to a request with a cached response.

## 5. Performance Evaluation

In this section, we present and discuss the evaluated performance of the CoAP proxy in a real implementation. The tests consider short and long lived communications. In both situations, we evaluated the CoAP proxy according to the use of WebSocket and HTTP long-polling. This allows gaining insight into the performance that the CoAP proxy can achieve with both protocols and evaluating which is the best option to use according to the kind of data transaction.

In long-lived communications, the CoAP proxy uses the observe protocol to receive updates from the CoAP device. A long-lived communication ends when the Web client receives 10 observe updates. In the short-lived case the request sent by the Web client implies a single response message from the CoAP proxy. Therefore A short-lived communication ends after the Web client receives the response.

The HTTP and WebSocket requests sent by the Web client, and the CoAP requests sent by the CoAP proxy use the GET method. Furthermore, CoAP requests and observe updates are sent as confirmable (CON) messages. These are used in CoAP to provide end-to-end reliability. In this sense, the destination node must acknowledge the reception of a CON request or update. The response or observe update sent by the CoAP device contains a data payload composed by a sequence of bits of fixed size. The composition and length of the CoAP request and response are specified in Table 1.

Crossbow's TelosB motes [24] are the hardware platform used to develop the test-bed network. TelosB is a typical example of a low-cost wireless sensor used in constrained sensor networks. It features 16-bit RISC MCU at 8 MHz and 16 registers. The platform offers 10 KB of RAM, 48 KB of flash memory and 16 KB of EEPROM.

**Figure 11.**
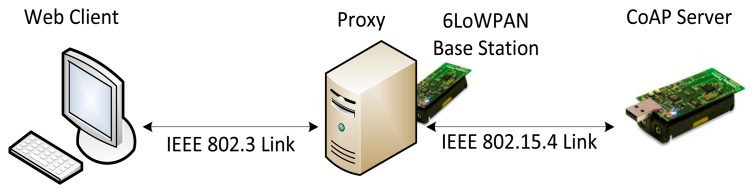
Test-bed network.

Our experiments are focused to evaluate simple short and long lived communications between the Web client, the CoAP proxy and the CoAP device. Thereby, the test-bed network can be kept simple and avoid deploying complex architectures. In this sense, we implemented a single-hop WSNs. The test-bed network is shown in Figure 11. The Web client and the CoAP proxy are located in two different PCs. These feature 2 GB of RAM and use Linux as operating system.

The proxy sends CoAP requests through a 6LoWPAN base station attached to its USB port. The CoAP device and the 6LoWPAN base station are embedded in TelosB motes. The CoAP device is located one-hop away from the base station. We used TinyCoAP [25] as the CoAP implementation embedded in the CoAP server.

Our study evaluates two parameters. First the RAM and ROM footprints of the CoAP proxy are evaluated. This analysis helps to evaluate the code complexity and the efficiency in terms of memory that the proxy has at run-time. Then, the latency is measured in order to evaluate the proxy response time. For all the tests, the results are reported according to the number of Web clients that simultaneously request data from the CoAP device. As mentioned before, the CoAP proxy uses the observe protocol to deal with long-lived communications. In this case, the CoAP device sends observe updates each 500 ms. This value is consistent with the update frequency of typical sensor monitoring in urban interaction processes in Smart Cities environments. The proxy establishes an observe relationship with the CoAP device when the first Web client requests it. The first notification sent to the subsequent observers is the value of the observed resource that is currently in the cache.

### 5.1. Memory Footprint

The CoAP proxy is designed to be embedded in any kind of hardware. However, its resource consumption has to be minimized to allow its use also in constrained hardware. In particular, the RAM memory used by the proxy at run-time has to be tailored to the characteristics of constrained devices where the available RAM could be in the order of few tens of megabytes. The analysis of the ROM footprint allows evaluating the complexity of the code that implements the COAP proxy. The analysis of the executable files sizes that are generated by the main proxy, Lighttpd and 6LoWPAN processes allow to evaluate of the ROM footprint. The ROM footprint of the CoAP proxy is therefore the sum of the size of each executable. The evaluation of the 6LoWPAN takes into account the memory that the CoAP proxy allocates to communicate with the 6LoWPAN interface. It does not consider the memory allocated by the 6LoWPAN interface. It is, in fact, embedded in a TelosB mote, which make impossible the use of valgrind profiling tool. Table 2 shows the results of the ROM memory consumption evaluation. The size of the ROM footprint is equal to 1466.9 KB, which is compliant with the characteristics of embedded applications.

**Figure 12.**
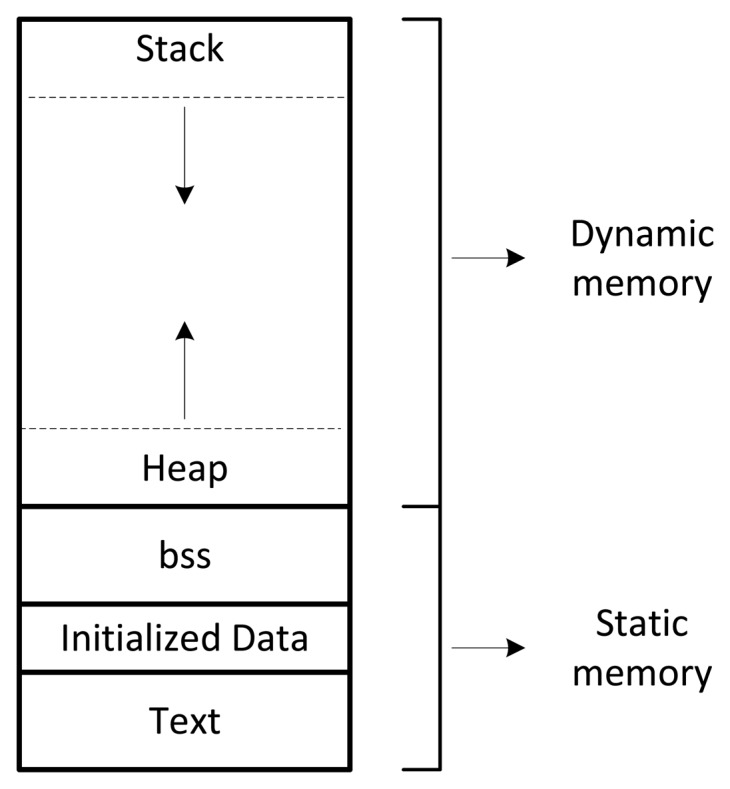
Layout of RAM memory.

The modules that compose the CoAP proxy are implemented using the C language. Therefore, the RAM footprint is analyzed according to the typical layout of a C program, which is shown in Figure 12. At run-time, memory is allocated dynamically in the heap and stack fields of the RAM using the malloc() and free() functions. The valgrind profiling tool is used to evaluate the size of the memory allocated dynamically at run-time. The text, bss and data_segment fields compose the part of the RAM that is allocated statically. Therefore, they have fixed size that do not vary at run-time. The text field contains the instructions that rule the execution of the program. The bss is used to store the variables that are not initialized. Finally, the data_segment contains the variables that have an initialization value. The static components of the RAM memory are evaluated using the size() command.

The RAM footprint is evaluated according to the number of Web clients that simultaneously request the representation of a resource or receive updates as observers. We evaluate short-lived as well as long-lived data transactions. The concurrency level is kept low considering the presence of 2, 10 and 50 simultaneous clients, which are enough to show the difference in terms of memory between WebSocket and HTTP long-polling. The variation of the concurrency level only affects the size of the memory allocated dynamically.

**Figure 13.**
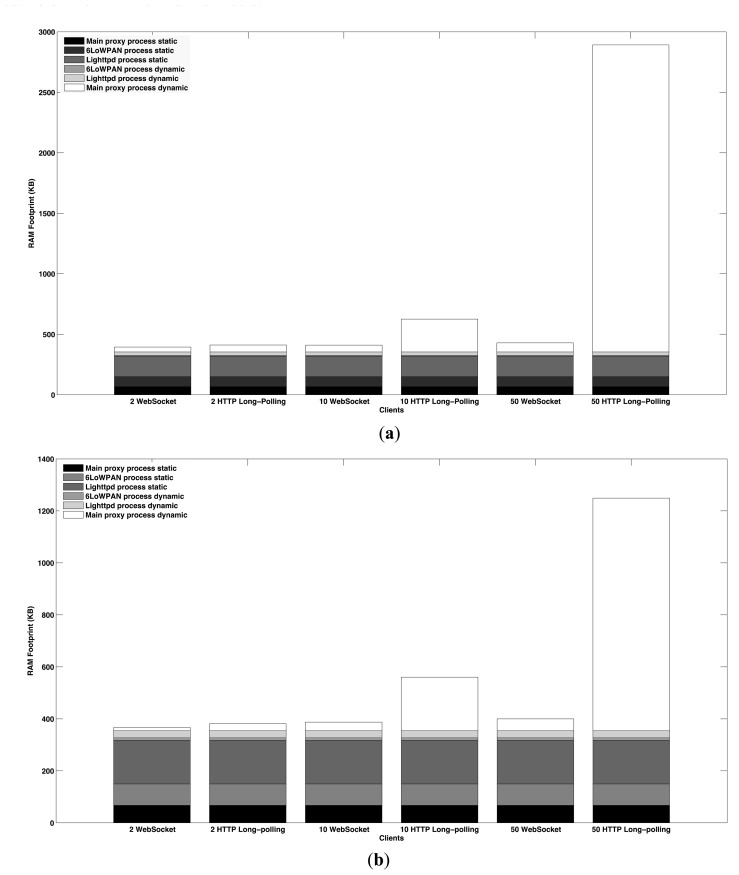
RAM footprint of the CoAP proxy. It has a low memory footprint when using WebSocket. The FastCGI protocol used in HTTP long-polling requires more complexity that results in a growth of the memory consumption. (**a**) RAM footprint of CoAP proxy in short-lived communications; (**b**) RAM footprint of the CoAP proxy in long-lived communications.

The RAM footprint is evaluated measuring the memory allocated by the static and dynamic components of each of the processes composing the proxy. The sum of the memory consumed by each process is the resulting size of the RAM footprint. The results are differentiated according to the use of Websocket and HTTP long-polling. The memory consumption of the Lighttpd process also is included in the WebSocket case. This process, in fact, is always present at run-time unless it is used only in HTTP long-polling. In this test, the RD is composed by a single node entry and the relative resource description. The cached response and the observer list are present only in the long-lived case. Figure 13 shows the results of the RAM evaluation tests.

The CoAP proxy has a lower RAM footprint with long-lived communications. The use of the observe protocol allows reducing the size of the dynamic memory that the main proxy process allocates to create and sends CoAP requests. The CoAP proxy, in fact, establishes only a single observe relationship with the CoAP device. This implies that the main proxy process has to allocate only the memory required to translate, create and send a single CoAP observe request and to acknowledge the subsequent updates. Further memory is allocated to send the updates to Web clients, to update the cache memory and to create and maintain the RD. In the short-lived case, instead, the CoAP proxy sends a CoAP request for each WebSocket or HTTP long-polling request that it receives. The main proxy process allocates, therefore, dynamic memory for each of these CoAP requests. Further memory is allocated to store the responses sent by the CoAP device and to send the relative acknowledgments.

In both cases, The CoAP proxy yields a better performance using WebSocket instead of HTTP long-polling. The handling of WebSocket requests, in fact, implies less complexity and overhead than that required for HTTP long-polling requests. In this sense, we found the use of the FastCGI protocol as the main cause of the high consumption reached by HTTP long-polling. The FastCGI requests intercepted by the Lighttpd server are multiplexed into a single connection and are processed by the main proxy process in a multi-threaded style. In that way better performance can be achieved with respect to creating a single thread to process each request. However, the presence of several concurrent processes increase the memory consumed by FastCGI. The dynamic memory allocated by the Lighttpd process does not vary with the concurrency level. As mentioned before, the Lighttpd server has a very low memory footprint that made it suitable for being embedded in constrained devices. The memory footprint of the Lighttpd server will increase only with a higher concurrency level.

In the Websocket case, the RAM footprint of the CoAP proxy is dominated by the memory allocated statically. The slight increase of the RAM footprint is due to the growth of the concurrency level. This has effect only on the memory allocated dynamically by the main proxy process to store the information related to each WebSocket requests.

The size of the dynamic memory allocated by the 6LoWPAN process is constant. The 6LoWPAN interface, in fact, receives by the main proxy process only a request at a time. The subsequent CoAP request is sent to the interface only when the previous has been sent. The dynamic memory allocated to create and send the 6LoWPAN packet is therefore constant.

### 5.2. Latency

**Figure 14.**
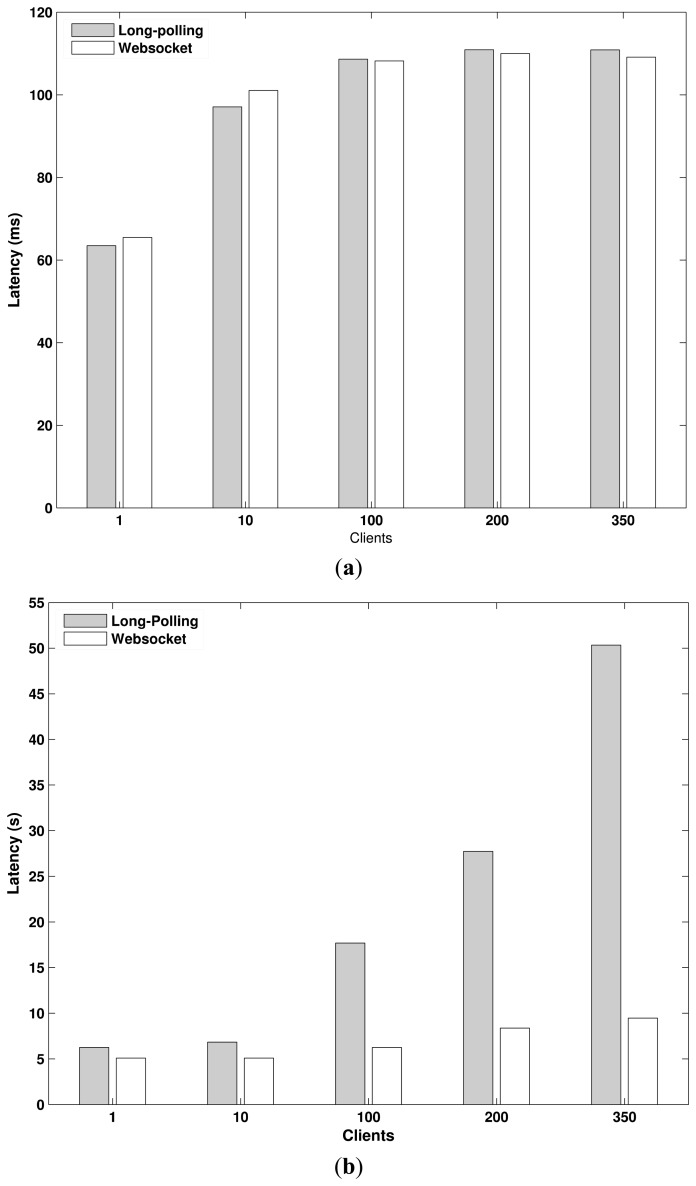
Latency for short and long lived communications. The CoAP proxy benefits from the use of WebSocket in long-lived communications. (**a**) Latency for short-lived communications; (**b**) Latency for long-lived communications.

The latency experienced by a Web client to retrieve data from the CoAP device is perhaps the most important parameter used to evaluate the goodness of the CoAP proxy implementation. In short-lived communications, latency is defined as the time elapsed from the moment the Web client sends a request until the moment it receives the response. The latency considers the delay introduced to establish and close the related TCP connection. In the long-lived case, the latency is the time elapsed from the moment the Web client requests to observe a resource until the moment it receives 10 updates. The latency, therefore, measures the length of the entire data transaction and not the sending of single updates. In fact, measuring the latency of each single update would not allow to highlight the difference between WebSocket and HTTP long-polling in long-lived data transaction. Instead, it would measure the latency as if the data transaction were short-lived. In addition, in this case we included the delay experienced to establish and close the TCP connection. Figure 14 shows the latency for each tested solution.

In long-lived communications, the CoAP proxy yields a better performance with WebSocket. In this context, the use of a persistent connection and the low overhead caused by the WebSocket protocol allows for reducing significantly the latency. The use of HTTP long-polling allows an acceptable performance when the level of concurrency is low. Instead, the latency undergoes a sharp rise when this level grows. In this case, the CoAP proxy is subject to a high workload that causes a rapid worsening of the performance. The high rate at which the Web clients requests the observe updates is the cause of this high workload. The use of the WebSocket protocol, instead, reduces significantly this workload. Consequently, the growth of the concurrency level causes only a small increase of the latency.

In short-lived communications, the CoAP proxy has almost the same performance with WebSocket and HTTP long-polling. The use of HTTP long-polling allows a slight improvement for low concurrency values. The difference between the performance of WebSocket and HTTP long-polling is mainly due to the delay introduced by the opening and closing handshake phases of the WebSocket protocol. However, this is partially compensated by the delay caused by the Lighttpd module to handle HTTP long-polling requests. This delay becomes predominant when the concurrency level is high causing a slight deterioration of the performance. WebSocket requests, instead, are received directly by the main proxy module, which allows reducing the delay caused by their handling.

## 6. Conclusions

This paper presents the design of a CoAP proxy [26] able to interconnect Web applications to CoAP devices located in WSNs, in the field of the smart energy grid, building and home automation, e-health, Smart Cities and intelligent transport systems. Its design is consequent to the analysis of the communication patterns that are used by the CoAP proxy to communicate with the CoAP device and the Web application. In this sense, considerate is considered the use of protocols able to deal with short and long lived communications.

The data flow caused by long-lived communications requires adopting protocols able to reduce the overhead and the workload that it could cause. The WebSocket protocol is included in the CoAP proxy to establish persistent connections with the Web applications. The observe protocol is used for the same purpose but to communicate with the CoAP device. The CoAP proxy also supports Web applications that use the traditional HTTP long-polling technique.

The design of the CoAP proxy includes a cache memory and a RD repository. The RD is used to host the description of the resources offered by the CoAP device. Its presence allows for simplifying the resource discovery process. Caching, instead, allows for reducing the number of messages interchanged between the CoAP proxy and the CoAP device. We also defined a convention format for the URI that should be used by a Web application to access a CoAP resource. The proposed format is compliant with the design principles of the RD and allows simplifying the translation process.

The performance of the CoAP proxy is evaluated considering long and short lived communications. In both cases the CoAP proxy is evaluated according to the use of HTTP long-polling and WebSocket. The performance evaluation has been done in terms of latency and memory consumption. Results show that, in long-lived communications, the CoAP proxy yields a better performance using the WebSocket protocol. Its use allows minimizing the communication overhead between the Web application and the CoAP proxy, which enhance the latency and RAM footprint performance. HTTP long-polling, instead, causes a high workload that results in a worsening of the performance especially for high concurrency levels. In the short-lived case, however, HTTP long-polling shows a good performance in terms of latency and, respect to WebSocket, reduces slightly the latency when the concurrency level is low. For higher levels, the CoAP proxy has almost the same latency performance with both protocols with WebSocket having a slight better performance. The CoAP proxy has a lower RAM footprint when using the WebSocket protocol. In this case, the FastCGI protocol used in HTTP long-polling entails further complexity to manage the HTTP connections that results in a high demand of RAM. The observe protocol proves to be able to reduce the RAM footprint of long-lived communications by reducing the messages interchanged between the CoAP proxy and the CoAP device.

In conclusion, the design proposed for the CoAP proxy offers an effective solution for interconnecting Web applications to CoAP devices. The use of different communication patterns provides flexibility and allows the CoAP proxy to work in different application scenarios. In particular, the inclusion of the WebSocket and the observe protocol allows improving the performance of long-lived applications. HTTP long-polling, instead, enables the use of the proxy in applications where short-lived communications are most used and the concurrency level is low.

## Figures and Tables

**Table 1.** Composition and length, in Bytes, of the messages interchanged between the CoAP proxy and the CoAP device.

**CoAP Request**	**Observe Request**	**CoAP Response**	**Observe Update**
CoAP Header: 5 B	CoAP Header: 5 B	CoAP Header: 5 B	CoAP Header: 5 B
Token: 1 B	Token: 1 B	Token: 1 B	Token: 1 B
URI: 5 B	Observe option: 2 B	Payload: 5 B	Observe option: 2 B
	URI: 5 B		Payload: 5 B

**Table 2.** ROM occupation of the CoAP proxy. Three modules compose the proxy.

**Main Proxy**	**Lighttpd**	**6LoWPAN**
302 KB	805.2 KB	359.7 KB
